# Subtotal parathyroidectomy for secondary renal hyperparathyroidism: a 20-year surgical outcome study

**DOI:** 10.1007/s00423-016-1447-7

**Published:** 2016-05-27

**Authors:** Aleksander Konturek, Marcin Barczyński, Małgorzata Stopa, Wojciech Nowak

**Affiliations:** Department of Endocrine Surgery, 3rd Chair of General Surgery, Jagiellonian University College of Medicine, 37 Prądnicka Street, 31-202 Krakow, Poland

**Keywords:** Secondary renal hyperparathyroidism, Subtotal parathyroidectomy, Intraoperative iPTH assay

## Abstract

**Aim:**

The aim of this study was to evaluate the outcomes of surgery for patients with secondary renal hyperparathyroidism (rHPT).

**Methods:**

This is a retrospective cohort study. Our institutional database was searched for eligible patients treated in 1995–2014. The inclusion criterion was initial parathyroidectomy for rHPT. Clinical and follow-up data were analyzed to estimate the cure rate (primary outcome), and morbidity (secondary outcome).

**Results:**

The study group comprised 297 patients (154 females, age 44.5 ± 13.7 years, follow-up 24.6 ± 10.5 months), including 268 (90.2 %) patients who had underwent subtotal parathyroidectomy, and 29 (9.8 %) who had had incomplete parathyroidectomy. Intraoperative iPTH assay was utilized in 207 (69.7 %) explorations. Persistent rHPT occurred in 12/268 (4.5 %) patients after subtotal parathyroidectomy and 5/29 (17.2 %) subjects after incomplete parathyroidectomy (*p* = 0.005). The patients operated on with intraoperative iPTH assay had a higher cure rate than non-monitored individuals, 201/207 (97.1 %) vs. 79/90 (87.8 %), respectively (*p* = 0.001). In-hospital mortality occurred in 1/297 (0.3 %) patient. The hungry bone syndrome occurred in 84/268 (31.3 %) patients after subtotal parathyroidectomy and 2/29 (6.9 %) subjects after incomplete parathyroidectomy (*p* = 0.006). Transient recurrent laryngeal nerve paresis occurred in 14/594 (2.4 %) and permanent in 5/594 (0.8 %) nerves at risk.

**Conclusions:**

Subtotal parathyroidectomy is a safe and efficacious treatment for patients with rHPT. Utilization of intraoperative iPTH assay can guide surgical exploration and improve the cure rate.

## Introduction

The most common consequence of chronic renal failure is represented by abnormalities of calcium-phosphate metabolism, leading to the patient developing secondary hyperparathyroidism. High phosphate levels, low or normal calcium concentration values, and vitamin D deficit play the fundamental role in the pathogenesis of the disease. Disturbances of the mutual correlations of the above factors result in uncontrolled, diffuse proliferation of parathyroid cells (polyclonal proliferation) in the initial stages of the disease, and subsequently in nodular proliferation (monoclonal proliferation) in patients with higher stages of chronic kidney disease [1–5]. The introduction of chronic dialysis programs, administration of vitamin D-derived, and phosphate-binding pharmaceuticals, as well as medications that regulate the activity of the calcium receptors through their continuous activation (calcimimetic agents) have markedly improved the situation of this group of patients. Unfortunately, as the disease persists, the population of monoclonal cells increases, what on the one hand leads to a progressive decrease in density of the calcium and vitamin D receptors, and on the other—to a maintained, stimulatory effect of phosphates on the parathyroid cells. Such a situation may pose an additional threat to the cardiac muscle and the entire circulatory system [6–10]. According to the data from the literature on the subject, between 15 % and approximately 40 % of patients require dialysis therapy and its duration is often positively correlated with the insensitive to conservative therapy nodular (monoclonal) type of parathyroid proliferation [11–13]. In such cases, a factor that plays a decisive role in the therapeutic success is a properly planned and executed surgical procedure, which—through interrupting autonomous PTH release allows for a possibility of regulating the level of phosphates and calcium as well as provides creates favorable conditions for a kidney transplant [14].

Among the contemporary surgical methods employed in secondary renal hyperparathyroidism, one should mention the following: subtotal parathyroidectomy (sPTX), where three glands and three fourths of the fourth are excised; total parathyroidectomy with autotransplanation parathyroid fragment of one gland to the sternocleidomastoid or brachioradial muscle (TPTX + AT); total parathyroidectomy without autotransplanation (TPTX); and total parathyroidectomy without autotransplanation and with bilateral cervical thymectomy (TPTX + BCT). From the viewpoint of the patient, the most important goal of all modalities of treating hyperparathyroidism, including surgical treatment, is improvement of quality of life. Thus, the overall goal is resolution of symptoms of hyperparathyroidism, at the same time ensuring normal calcium and phosphorus metabolism in patients with end-stage kidney disease. The strategy of surgical treatment should thus strive for an appropriate balance between the extent of parathyroidectomy, prevention of recurrent disease, and avoidance of postoperative permanent hypoparathyroidism [14–21].

The objective of the present paper based on the material collected by the Department is an analysis of results achieved in treating secondary renal hyperparathyroidism based on the method of subtotal parathyroidectomy and employing intraoperative iPTH monitoring, the operations having been performed within the past 20 years.

## Materials and methods

In our publication, we made an attempt at presenting the inclusion criteria of surgical treatment based on recommendations issued by The National Kidney Foundation Kidney Disease Outcomes Quality Initiative and Kidney Disease: Improving Global Outcomes (KDIGO). The KDOQI guidelines suggest: “… maintaining iPTH levels between two to nine times the upper normal limit for patients in dialysis. Surgery may further be indicated for patients with uncontrollable renal HPT while medical treatment with phosphate binders, calcium supplements, active vitamin D analogues and cinacalcet expected long-term survival with severe symptomatic renal HPT including pruritus, intractable bone pain, advanced osteopenia/osteoporosis, calcinosis and calciphylaxis.” [14].

Based on a retrospective analysis of data originating from the clinical register of parathyroid procedures performed in the years 1995–2014, the assessment included a group of 297 patients operated on due to secondary renal hyperparathyroidism in a single referral clinical center. Analyzing the above patient’s group operated on in our center we noted the duration of the chronic kidney disease ± SD being 139.9 ± 80.7 months and duration of dialysis (mean ± SD)—88.6 ± 106.1 months. The above data originated from medical records provided by dialysis units. In turn, based on a retrospective analysis originating from the our clinical and ambulatory surgery registers, we observed that well-documented data were within the range of 1 month to more than 4 years and calculated the mean value as more than 24 months. The analysis took into consideration data derived from the medical history and physical examination of the patients, results of pre- and postoperative biochemical diagnostic and imaging tests, data from surgical protocols, out-patient follow-up records, and histology results. Clinical characteristic of the investigated group is presented in Table 1.

**Table 1:** Clinical characteristics of 297 patients with renal HPT qualified for operation in the years 1995—2014

Age, mean ± SD (years)	44.5 ± 13.7
Sex ratio (M:F), number	154/143
Duration of the chronic kidney disease ± SD (month)	139.9 ± 80.7
Duration of dialysis mean ± SD (month)	88.6 ± 106.1
Weight of removed parathyroid gland per patient; mean ± SD (g)	4.53 ± 1.5
Intrathymic localization number (%)	64 (21.5)
Causes of renal HPT	
Glomerular and tubular kidney disease:	
a) primary: focal segmental glomerulosclerosis immunological nephropathy number (%)	203 (68.3)
b) polycystic kidney disease number (%)	27 (9.1)
c) secondary: diabetic nephropathy number (%)	12 (4.1)
Another kidney disease:	
e.g.: lupus nephritis, primary and secondary amyloidosis, bilateral renal artery stenosis number (%)	55 (18.5)
Comorbid diseases and signs:	
hypertension number (%)	232 (78.1)
Cardiac diseases:	
e.g.: coronary artery disease, cardiac dysrhythmia, myocardial infarcaction number (%)	77 (25.9)
Digestive tract diseases:	
e.g.: peptic ulcers, cholelithiasis number (%)	69 (23.2)
soft tissue calcification number (%)	63 (21.2)
itching of the skin, muscle weakness, ostalgia	241 (81.1)
Comorbid nodular goiter number (%)	31 (18.6)
Time of hospitalization (days) (mean ± SD)	4.9 ± 2.5
Mean follow-up (months) (mean ± SD)	24.6 ± 10.5

The analyzed group of patients met the following criteria:

- in all the patients, the cause of secondary hyperparathyroidism (SHP) was end-stage renal failure (RN),
- all the patients were on renal dialysis in keeping with a defined protocol, being dialyzed not less frequently than three times per week,
- biochemical confirmation of SHP was based on elevated PTH concentration levels >500 pg/ml (N = 12–65 pg/ml), hyperphosphatemia (N = 0.81–1.62 mmol/l); the values of calcemia (N = 2.2–2.6 mmol/l) and the Ca × Pi quotient > 5. 6 mmol^2^/l^2^ (Ca × P q >70 mg%) (N < 4.4 mmol^2^/l^2^),
- resistance to conventional therapy (calcimimetics, vitamin D and calcium preparations, medications decreasing intestinal phosphate absorption),
- intensified clinical symptoms (regardless of serum Ca concentration levels), ostalgia and arthralgia, pruritus, pathological fractures, metastatic calcifications and vascular calcifications, signs of calciphylaxis.

Preoperative localization diagnostic management has included ultrasound of the neck, which since 2003 has been performed by the operating surgeon, regardless of the results of ultrasonography performed elsewhere. Subtraction scintigraphy of the parathyroids was initially performed using the technetium-thallium technique (99 m Tc + 201 Tl scintigraphy), and subsequently by the technetium-MIBI (methoxyisobutylisonitrile) scan (sestaMIBI:99 m Tc MIBI scintigraphy); the imaging was done outside our center prior to referring the patient for surgical treatment. In the analyzed material, in all the reoperated patients, the diagnostic method of choice was scintigraphy (sestamibiscan) combined with another complementary imaging study based on magnetic resonance (NMR). The surgical technique included bilateral neck exploration (BNE) performed by experienced surgical team (in the Department where the authors are employed, approximately 1000 thyroid operations are performed annually, including approximately 110–130 parathyroidectomies-primary, secondary, and tertiary hyperparathyroidism). Having exposed the space around the thyroid lobes, the recurrent laryngeal nerves were visualized and subsequently, the sites of the most common location of all the four parathyroid glands were meticulously explored and their size was macroscopically assessed. The extent of the procedure included subtotal parathyroidectomy, beginning from excising three fourths of the parenchyma of the smallest gland (leaving intact an approximately 100 mg fragment with the lowest spectrum of pathologic changes and normal blood supply) and subsequently resecting the remaining enlarged and pathological parathyroid glands. Thymectomies were performed in all the patients, in whom visualization of all the glands was not possible; since 2003, they have become standard procedures performed in the course of parathyroidectomy. In cases of concomitant thyroid pathology, depending on the extent of nodular lesions, lobectomy or total thyroidectomy was performed in a one-stage procedure. Initially, the effectiveness of the surgical procedure was assessed based on intraoperative histology (up to 2008). At the end of 2002, the Department represented by the authors introduced a fast intraoperative iPTH determination method (STAT-IntraOperative-Intact-PTH Immunoassay; Future Diagnostics, Wijchen, The Netherland) to be employed in blood samples preoperatively, at 10 and 20 min after subtotal parathyroidectomy. The criterion of procedure effectiveness was assumed to be represented by a drop in iPTH concentration value following parathyroid excision by 60 % after 10 minutes and by at least 80 % after 20 min as compared to the initial (preoperative) value. A decrease lower than the above-mentioned percentages prompted a search for a supernumerary gland or ectopic parathyroid tissue and was a prognostic factor for a failure of surgical treatment [17]. The criterion of persistent hyperparathyroidism was accepted as absence of iPTH drop below 60 % of the initial value immediately after surgery and 300 pg/ml in late follow-up, or an iPTH increase within 6 months postoperatively in spite of initially observed normalization. The mean hospitalization time was 4.9 ± 2.5 days (1–18 days). Patients with significant postoperative hypocalcemia required administration of calcium preparations and introducing calcium-containing dialyzate to their renal dialysis program. The mean follow-up in the analyzed group was 24.6 ± 10.5 months (1–50 months). Indirect laryngoscopy is performed in each patient by an ENT specialist on day 1 postoperatively and calcium levels were determined, assuming hypocalcemia to occur at total calcium levels below 2.0 mmol/l. In our Department, the patients have been divided into three groups, depending on serum calcium concentration values; thus, we have distinguished between patients with mild (2.0–2.19 mmol/l), moderate (1.8–1.99 mmol/l), and severe (<1.8 mmol/l) hypocalcemia.

The statistical analysis was performed employing the STATISTICA software (version 10, StatSoft, Poland). Assessment of the variability of the investigated variables was performed by means of arithmetic means, standard deviation (SD), minimum and maximum values (min-max), and prevalence rates expressed as percentages.

An inter-group comparison of particular properties was performed by the student’s *t* test for variables with normal distribution and by the χ square test for the remaining variables.

The significance level was assumed to be α ≤ 0.05.

## Results

In the analyzed group of 297 patients, no significant differences were noted between the number of females and males (143 vs. 154), while the mean age was 44.5 ± 13.7 years (16–79 years). The mean follow-up time was slightly above 2 years (24.6 ± 10.5 months). The duration of hemodialysis treatment, although being within a fairly wide range of 2 to 20 years, was approximately 7 years on the average (88.6 ± 106.1 years). In turn, the mean duration of chronic kidney disease treatment did not exceed 12 years (139.9 ± 80.7). The most common cause of renal failure (81.5 %) was primary or secondary glomerular or tubular damage. In the remaining cases (18.5 %), renal failure developed with other disease entities as the background, with systemic autoimmune diseases and vascular lesions predominating.

Among concomitant diseases, the most numerous group included patients with arterial hypertension and diseases involving the coronary vessels and cardiac muscle (78.1 and 25.9 %, respectively). Sixty-three patients (21.2 %) presented with calcium deposits deposited in the soft tissues, mainly in the subcutaneous tissue of the lower and upper extremities, both in the straight fragments of the limbs and in the joint regions. Concomitant thyroid pathologies manifested as non-toxic nodular goiter were observed in 31 patients (18.6 %). The mean duration of hospitalization was approximately 5 days (4.9 ± 2.5). One patient died (1/297 = 0.3 %) (Table 1).

In the analyzed 20-year period, 268 (90.2 %) patients were subjected to subtotal parathyroidectomy; in the remaining group of 29 patients (9.8 %), the procedure was incomplete. In both the analyzed groups, the mean preoperative concentration values of PTH (sPTX group 1531.4 ± 647.8 versus incomp PTX group 1588.1 ± 609.7), calcium (sPTX group 2.56 ± 0.05 versus incomp PTX group 2.58 ± 0.02), and phosphates (sPTX group 2.41 ± 0.52 versus incomp PTX group 2.38 ± 0.38) did not differ.

Persistent hyperparathyroidism was noted in 12/268 (4.5 %) patients after subtotal resection of the glands and in 5/29 (17.2 %) patients in the second analyzed group. The values were statistically significant (*p* = 0.005). Intraoperative iPTH monitoring allowed for achieving a higher percentage of permanent cures in late follow-up in the group of patients with vs. without intraoperative iPTH monitoring (IOPTH group 201/207–97.1 % versus without iPTH group 79/90–87.8 %) (*p* = 0.001). The values were statistically significant.

In the group of patients after subtotal parathyroidectomy, a significant decrease was noted in phosphate levels 6 months after surgery as compared to the group of patients with incomplete parathyroid resection (sPTX group 1.48 ± 0.51 versus incomp PTX group 2.32 ± 0.41; *p* < 0.001).

A similar relationship was also observed with respect to the mean PTH concentration values in the two analyzed groups 6 months after the surgery (sPTX group 122.9 ± 182.2 versus incomp PTX group 344.5 ± 208.3; *p* < 0.001). Despite oral calcium and vitamin D supplementation (Calperos 1 g 3 × 2 tablets/day; Alphadiol 1 caps. μg/day), 84 patients after subtotal parathyroidectomy-manifested symptoms of intensified hypocalcemia and hypophosphatemia, what allowed for diagnosing the hungry bone syndrome. These patients required intravenous calcium administration and dialysis treatment with calcium-containing dialyzate. The above association was reflected in the statistically significant difference (*p* = 0.006) in the manifestation of symptoms of the hungry bone syndrome between the two analyzed groups (sPTX group 31.3 % (84/268) versus incomplete PTX group 6.9 % (2/29). There was noted difference in postoperative transient recurrent laryngeal nerve paresis. Transient unilateral paresis of the recurrent laryngeal nerve was noted in 6/58 (10.3 %; *p* < 0.001) patients in incomplete PTX group, while permanent paresis was seen in 3/58 (5.2 %) cases in the same group (in relation to 594 nerves at risk) (Table 2).

**Table 2:** Comparison of two groups of 297 patients undergoing subtotal PTX and incomplete PTX surgery with rHPT

	Subtotal PTX	Incomplete PTX	*P*
Number of patients (%)	268 (90.2)	29 (9.8)	NA
iPTH pg/ml (mean ± SD)			
preoperatively baseline	1531.4 ± 647.8	1588.1 ± 609.7	0.651
6 months postoperatively	122.9 ± 182.2	344.5 ± 208.3	<0.001
Average serum calcium level mmol/l (mean ± SD)			
preoperatively baseline	2.56 ± 0.05	2.58 ± 0.02	0.034
6 months postoperatively	2.31 ± 0.21	2.55 ± 0.01	<0.001
Average serum phosphorus level mmol/l (mean ± SD)			
preoperatively baseline	2.41 ± 0.52	2.38 ± 0.38	0.763
6 months postoperatively	1.48 ± 0.51	2.32 ± 0.41	<0.001
Persistent disease, number (%)	12 (4.5)	5 (17.2)	0.005
Hungry bone syndrome, number (%)	84 (31.3)	2 (6.9)	0.006
Unilateral RLN injury, number (%)^a^			
transient	8 (1.5)	6 (10.3)	<0.001
permanent	2 (0.4)	3 (5.2)	0.003

Reference range 12–65 pg/ml, calcium total 2.2–2.6 mmol/l; phosphate 0.81–1.62 mmol/l

*NA* not available, *iPTH* parathyroid hormone

^a^RLN injury was calculated for nerves at risk and not for patients (there were 594 nerves at risk in all patients with rHPT group)

**Fig. 1:**
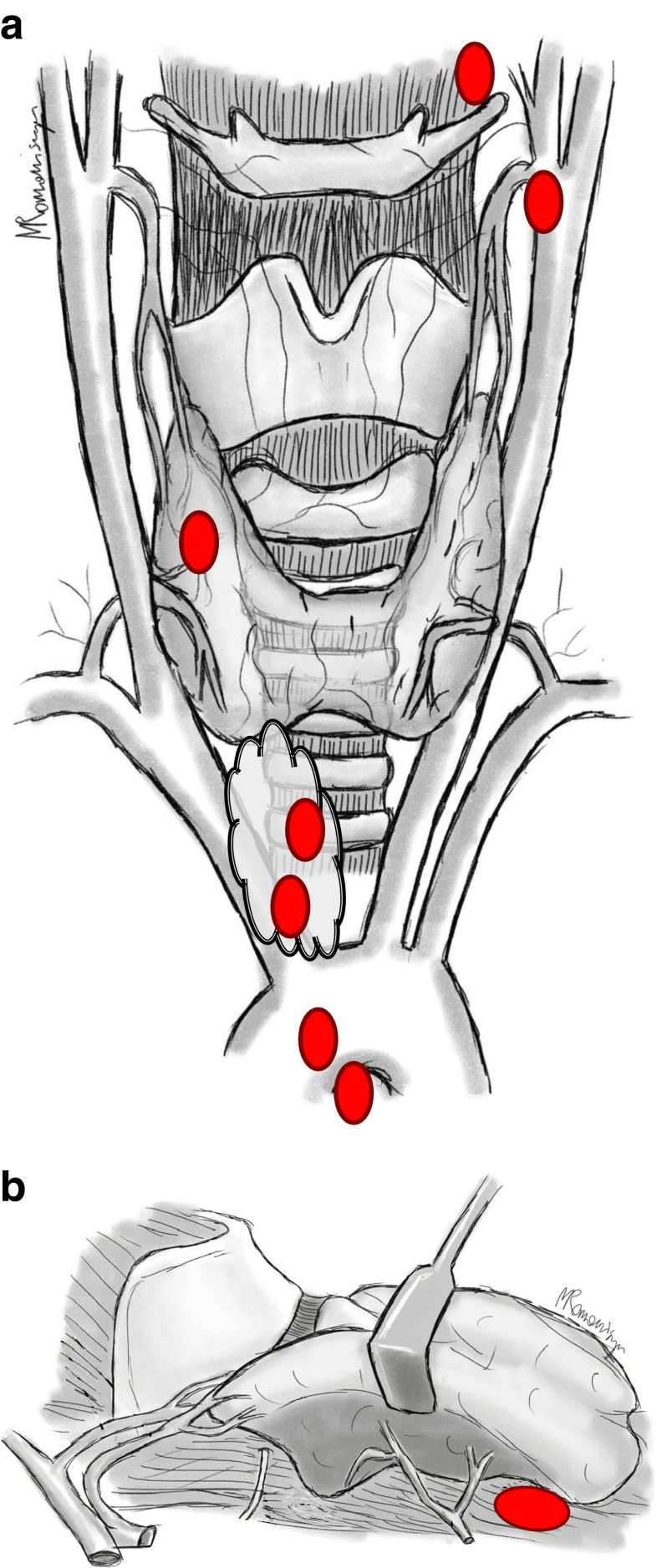
**a** Localization of the resected ectopic parathyroid gland in patients with rHPT (*anterior-posterior view*). Localization of ectopic and supernumerary parathyroid glands: mediastinal outside thymus, retroesophageal space, intrathymic, intrathyroid, near hyoid bone, and near carotid sheath. **b** Localization of the resected ectopic parathyroid gland in patients with rHPT (*lateral view*). Localization of ectopic gland: retroesophageal space

**Fig. 2:**
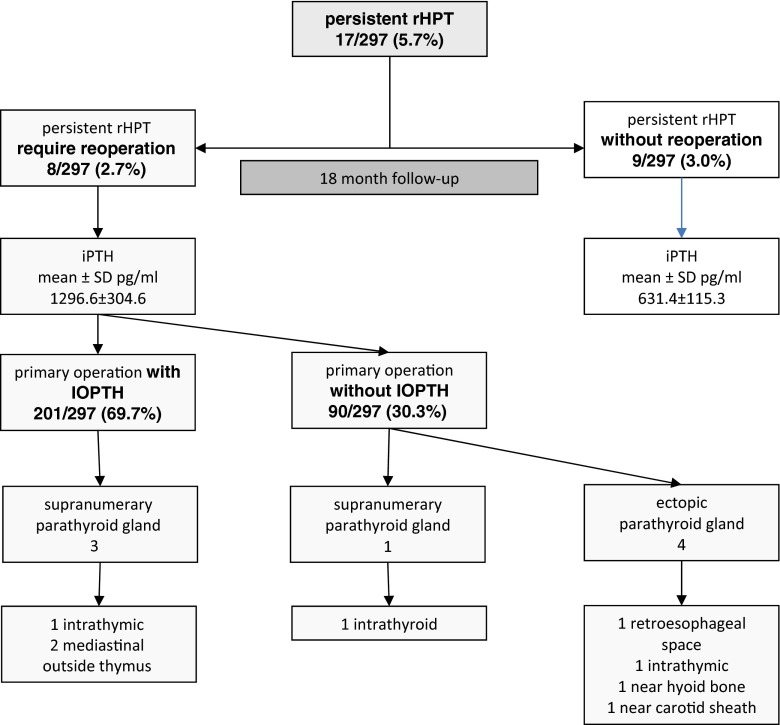
Localization parathyroid tissues in group of the patients require reoperations with persistent rHPT

In 17 patients with persistent hyperparathyroidism, elevated PTH values were noted at follow-up 6 months postoperatively (after 6 months 567.3 ± 234.2 pg/ml). Nevertheless, only eight patients from this group showed the mean PTH values amounting to 1296.6 ± 304.6 pg/ml 18 months postoperatively; *p* < 0.001 (Table 3). The patients needed reoperation. The cause of persistent hyperparathyroidemia in the group with intraoperative PTH concentration monitoring was the presence of three supernumerary parathyroid glands, while in the group without PTH determinations—the presence of one supernumerary parathyroid gland and four ectopically situated parathyroids. The precise location of the reoperated parathyroid glands and PTH levels is presented in Fig. 1a, b and Fig. 2.

**Table 3:** Mean iPTH serum levels in the group patients with unsuccessful first operation in rHPT (persistent rHPT)

	Persistent rHPT requiring reoperation (*n* = 8)	Persistent rHPT without reoperation (*n* = 9)	*P*
Time resection	iPTH ± SD	iPTH ± SD	
Preoperative baseline	1563.9 ± 485.8	1397.3 ± 459.5	0.479
Follow-up			
6 months after first operation	721.1 ± 239.6	413.4 ± 70.3	0.002
18 months after first operation	1296.6 ± 304.6	631.4 ± 115.28	<0.001

## Discussion

A consequence of renal failure is the patient developing secondary hyperparathyroidism. As it follows from the reports of the European Society of Dialysis and Transplantation, the duration of dialysis treatment is a factor affecting qualification of patients to parathyroidectomy. A decision to choose a treatment modality (surgical/conservative) always requires a discussion among members of an interdisciplinary team, most commonly including nephrologists, surgeons, anesthesiologists or cardiologists. As we know, the introduction of calcimimetics has resulted in a significant decrease in the number of patients referred for surgical treatment; nevertheless, in keeping with data from the world literature, it is assumed that each year, approximately 1–2 % of patients with renal hyperparathyroidism require parathyroidectomies [10, 20–23]. In the presently analyzed material, the mean age of the patients, the duration of renal failure, and the mean duration of dialysis treatment did not fundamentally differ from data reported in the literature on the subject. The principal cause of the developing renal failure is believed to be represented by primary diseases of the glomeruli and tubules, as well as autoimmune disorders, which lead to secondary destruction of renal parenchyma. In the present material, in 86.8 % patients the lesions developed in the presence of the above underlying diseases and only in less than 5 % (4.1 %), diabetic nephropathy was responsible for renal failure. The above data approximate findings from the literature; where at present, diabetic nephropathy accounts for a low percentage of causes of CRF; what is an evidence of progress having occurred over the years in proper management of diabetic patients [11–13, 20, 21].

Progressive kidney damage leading to destruction of nephron function is associated with abnormal functioning of the feedback axis associated with calcium and phosphate ions. Nevertheless, the key role is ascribed here to phosphates, the level of which is regulated by well-functioning proximal renal tubules [12, 13, 22]. Thus, concentration values of PTH and phosphates play a decisive role in determining the intensity of various symptoms, and the very Ca × P quotient predominantly depends on their concentration values, in this way becoming a significant factor implicated in skin lesions, calcifications involving the soft tissues or deposition of calcified atherosclerotic plaques in the vascular walls.

Analyzing the present data in terms of the occurrence and intensity of skin symptoms, pruritus, ostalgia (81.1 %) as well as arterial hypertension (78.1 %) and other diseases of the circulatory system (25.9 %), we noted that they did not differ from the data from the literature, as the same time pointing to the role of high serum phosphate values in patients on dialysis treatment (Tables 1 and 2) [10].

In our Department, surgical treatment of hyperparathyroidism in patients with renal failure is based on subtotal parathyroidectomies, in view of the fact that their advantages include preservation of hormonal function by the parathyroid stump with normal blood supply left in situ, creation of favorable conditions for postoperative serum calcium monitoring, and in case of patients awaiting a kidney transplant, taking into consideration duration of dialysis therapy, it provides favorable conditions for such a procedure, preventing damaging the transplanted kidney. The above-presented method of surgical treatment was employed in 268/297 (90.2 %) patients. The remaining group included individuals subjected to incomplete resection of the glands (29/297—9.8 %). In this group, 5/29 (17.2 %) cases of persistent hyperparathyroidism were noted, what—as compared to the number of patients presenting with persistent hyperparathyroidism (12/268—4.5 %) following subtotal parathyroidectomy—yielded a statistically significant result. The above data were comparable to the findings reported in the world literature on the subject [23–27]. As it follows from the human ontogenetic development, the said variability results from the variable origin of the superior and inferior parathyroid buds (III and IV pharyngeal folds) and their subsequent migration in the course of fetal maturation and growth. Failures in surgical treatment of secondary hyperparathyroidism are thus most commonly the result of missing a supernumerary (e.g., fifth) or ectopically situated parathyroid gland or an inadequate resection of the glands [20, 28, 29].

Irrespectively of the above-mentioned causes, such patients require treatment. Does it always has to be surgical treatment and is there a method allowing for a better assessment of prognostic factors predicting a success of operative treatment? While analyzing the period of past 20 years, the authors found out that the effectiveness and successfulness of the surgical procedure were affected by the method of prompt intraoperative iPTH monitoring introduced in the Department at the end of 2002. The criterion of operation effectiveness was a 60 % decrease in iPTH concentration value after 10 min and at least an 80 % decrease 20 min postparathyroidectomy as compared to the initial (preoperative) value. A drop lower than the above-mentioned iPTH concentration values favored a search for supranumerary or ectopic parathyroid tissue and was a prognostic factor of an operative failure—a higher permanent cure rate as compared to patients without intraoperative iPTH monitoring (IOPTH group 201/207 (97.1 %) versus without iPTH group 79/90 (87.8 %), (*p* = 0.001)). The values were statistically significant [17, 18].

Special attention should be paid to a skilful interpretation of results based on the knowledge of the kinetics of PTH level decrease in a unit of time and the resultant implications addressing the occurrence of both falsely positive and falsely negative results. Looking back at the past 20 years of parathyroid surgery in our center, we do realize the important role of iPTH monitoring and of accuracy and ability to interpret the results and therefore make therapeutic decisions in severely ill patients with renal hyperparathyroidism. False positive results defined as a significant intraoperative iPTH decrease in a patient with persistent postoperative hyperparathyroidism occur on the average in 3–5 % of cases. In our Department: the criterion of surgery effectiveness is assumed to be a drop in iPTH concentration value by 60 % after 10 min and at least by 80 % after 20 min as compared to the initial value. Such iPTH values give a better guarantee that the result is not false positive. A drop lower than the above-mentioned iPTH values prompts a search for supranumerary or ectopic parathyroid tissue and is a prognostic factor of an operative failure. Another problem is posed by falsely negative results, defined as a non-significant drop in iPTH level, in spite of successful parathyroidectomy having been performed. The incidence of such results is lower as compared to false positive results and as a rule does not exceed 2 %. Such a result is affected by knowledge of the iPTH half-life (generally approximately 4 min on the average) and a pronounced increase of iPTH level during parathyroids preparation prior to their resection. Employment of the aforementioned protocol by the authors, i.e., collection of at least two blood samples for iPTH determinations at 10 and 20 min following parathyroids resection to a great extent eliminated the possibility of obtaining a false negative result caused by the very variability of iPTH kinetics (the period is several times longer than the iPTH half-life) [17]. As it has been mentioned, in the investigated group, 17 patients demonstrated persistent hyperparathyroidism in 5/29 (17.2 %) in the group subjected to incomplete parathyroidectomy and in 12/268 (4.5 %) of individuals after subtotal parathyroidectomy. According to the literature, the percentage covers a relatively broad range of 5–34 % [30–33]. The introduction of modern scintigraphic imaging methods (123I/99mTc-Sestamibi SPECT/single photon emission computed tomography/) and the use of magnetic resonance imaging (MRI) is of a great assistance in identification of supernumerary or ectopic parathyroid tissue. In the analyzed material, in all the reoperated patients, the diagnostic method of choice was scintigraphy (sestamibiscan) combined with another complementary imaging study based on magnetic resonance (NMR). It should be emphasized that while analyzing various imaging methods, one may perceive certain regularity—a larger parathyroid gland means a better chance for its visualization and by the same token for a successful surgical treatment. Based on the above observation, one may also surmise that the size and weight of parathyroid glands may be helpful in their identification during surgery. In the present material, the mean weight of the resected parathyroid glands was approximately 4.5 g [24, 34, 35].

Do all patients, however, require a reoperation? On the one hand, finding a parathyroid gland in location diagnostics and biochemical criteria of an operative failure is highly helpful in making the decision to reoperate. On the other hand, some authors are of the opinion that maintaining the hormone level that is two–fourfold higher than the normal value affects both the level of calcium and phosphates, what in combination with the knowledge of progressing with disease duration time resistance of bone tissue to parathormone and prevention of adynamic bone disease is of a paramount importance in quality of life of surgical patients. Therefore, some authors believe that sPTX is a better therapeutic method for dialyzed patients awaiting a kidney transplant, since overcoming PTH autonomy, it prevents damaging the transplanted kidney. This is also important for another reason: as it has been observed in patients with tertiary hyperparathyroidism, posttransplantation renal failure manifested as a higher decrease in GFR was higher in TPTX + AT patients as compared to patients after sPTX. In turn, total parathyroidectomies may be more frequently employed in patients who are not candidates for kidney transplants; nevertheless, it requires exercising much caution in postoperative management. This affects both the level of calcium and phosphates. Hypocalcemia and developing symptoms of the hungry bone syndrome are not solely the result of excessive incorporation of calcium to the osseous system, but also of the postoperative drop in phosphate levels. This is why monitoring of postoperative calcium values is justified only as a preventive measure of developing hypocalcemia with its uncomfortable symptoms, but it not always defines the effectiveness of surgical treatment. Hence, there emerges a long-term need for monitoring both the level of PTH and phosphates in patients subjected to parathyroidectomy. Normalization of the above parameters provides good foundations for a renal transplant, decreasing the number of early rejections [33, 36–38].

In the present material of 17 patients (5/29 incomplete PTX + 12/268 sPTX) with unsuccessful primary procedures, who manifested a PTH level increase 6 and 18 months postoperatively, reoperations were performed in eight subjects with PTH levels being significantly higher as compared to the remaining patients not qualified for reoperations (1296.6 ± 304.6 versus 631.4 ± 115.28; <0.001) (Table 3). The analysis of causes of persistent hyperparathyroidism demonstrated that in the group of reoperated patients with IOPTH (3/6 in the group of 207 patients with IOPTH), the main cause of high PTH concentration values 18 months after the primary procedure was supernumerary, ectopic parathyroid glands situated in the mediastinum and deep within the thymus, while in the remaining group of patients without IOPTH (5/11 of the group of 90 patients), ectopic abnormalities predominated, their location being variable and involving the neck and mediastinum (Fig. 1a and b), (Fig. 2) [10, 17–35, 39].

Although a retrospective analysis of the data demonstrates the effectiveness of a standardized operative method employing intraoperative PTH monitoring, the relatively short follow-up time in the analyzed group (24.6 ± 10.5) limits answering another question, i.e., whether renal failure affects changes in PTH metabolism and by the same token affects assessment of the effectiveness of surgery.

The analysis of the literature describing the results of all types of surgical treatment employed in secondary hyperparathyroidism does not provide us with unambiguous criteria of the selection and supremacy of a single given method. The ultimate goal is always resolution of hyperparathyroidism symptoms at the same time providing the patients with end-stage renal disease with a guarantee of normal calcium and phosphorus metabolism. Some authors believe that sPTX is a better therapeutic method for dialyzed patients awaiting a kidney transplant, since, breaking the PTH autonomy, it prevents damage to the transplanted kidney; in turn, total parathyroidectomy may be more frequently employed in patients who are not candidates to kidney transplants, but the modality requires exercising much caution in postoperative management. Summing up, based on numerous publications on surgical treatment of secondary hyperparathyroidism, it should be stated that both subtotal PTX and TPTX + AT and standard cervical thymectomy represent procedures that ensure a postoperative success with an acceptable and satisfactory percentage of permanent hypoparathyroidism [27, 33, 36–38, 40–44].

Both the advocates and opponents of particular therapeutic methods employed in secondary renal hyperparathyroidism agree that routine IOPTH becomes of assistance in determining the effectiveness of the operation with respect to its extent, is a helpful criterion in reaching a decision to complete the procedure, at the same time being a prognostic factor of treatment adequacy and in a situation when there is no significant drop in concentration values, it provides the surgeon with an opportunity to decide on further exploration of the surgical field to seek ectopic glands [40–44].

In the opinion of the authors, the decisive factors that affect the effectiveness of surgical treatment in rHPT is the experience of the surgeons and of the center whether treatment is provided, accessibility of intraoperative iPTH monitoring knowledge of mechanisms that regulate the calcium-phosphate metabolism and follow-up duration.
